# Age- Matched Comparison of Children Hospitalized for 2009 Pandemic H1N1 Influenza with Those Hospitalized for Seasonal H1N1 and H3N2

**DOI:** 10.1371/journal.pone.0021837

**Published:** 2011-07-20

**Authors:** Susan S. Chiu, Kwok-Hung Chan, Wilfred H. S. Wong, Eunice L. Y. Chan, J. S. M. Peiris

**Affiliations:** 1 Department of Paediatrics and Adolescent Medicine, The University of Hong Kong, Pokfulam, Hong Kong; 2 Department of Microbiology, The University of Hong Kong, Pokfulam, Hong Kong; 3 Hong Kong University Pasteur Research Centre, Pokfulam, Hong Kong; University of California Los Angeles, United States of America

## Abstract

**Background:**

A wide spectrum of clinical manifestation ranging from deaths to a mild course of disease has been reported in children infected with the 2009 pandemic H1N1 (pH1N1) influenza.

**Methodology/Major Findings:**

We conducted an age-matched control study comparing children hospitalized for pH1N1 with historic controls infected with seasonal H1N1 and H3N2 influenza to correct for the effect of age on disease susceptibility and clinical manifestations. We also compared children with pH1N1 to children concurrently admitted for seasonal influenza during the pandemic period to adjust for differences in health-seeking behavior during the pandemic or other potential bias associated with historic controls. There was no death or intensive care admission. Children with pH1N1 were more likely to have at least one risk condition for influenza, an underlying chronic pulmonary condition, more likely to have asthma exacerbation and to be treated with oseltamivir. There was no difference in other aspects of the clinical course or outcome.

**Conclusion:**

Disease manifestation of children hospitalized for pH1N1 infection was mild in our patient population.

## Introduction

Pandemic H1N1 (pH1N1) influenza emerged in March 2009 in Mexico. [1], [2] There have been case series reports of severe disease and deaths, including that in children. [1]–[9]. However, there are also reports of a wide spectrum of clinical manifestation including a mild course of disease. [10]–[15]. Recently, a few studies compared pH1N1 with seasonal influenza infection. One study showed that children with pH1N1 presented with more lower respiratory infections and gastrointestinal symptoms than children with seasonal influenza in the previous 2 years. [16]. Two studies using different cohorts for comparison showed that pH1N1 did not appear to cause more severe disease than seasonal influenza A in children. [17], [18]. However, none of these studies were age-matched and children of different age groups might have different susceptibility and disease manifestations. We therefore sought to document the characteristics and manifestations of children admitted to Queen Mary Hospital, Hong Kong with laboratory-confirmed pH1N1 and compared them to age-matched historic controls admitted to the same hospital with seasonal influenza. We also compared children hospitalized with pH1N1 with those concurrently admitted for seasonal influenza to correct for altered health seeking behavior during a pandemic and other potential biases associated with historic controls.

## Materials and Methods

Queen Mary Hospital is a tertiary-care public hospital and is the teaching hospital of The University of Hong Kong. The pediatric general ward and intensive care unit take admission from its emergency department as well as referrals from other hospitals and clinics. We have been routinely performing virologic testing on nasopharyngeal aspirate (NPA) samples on all children admitted with fever and signs of acute respiratory symptoms such as rhinitis, cough or sorethroat since 1995. We conducted a retrospective study by retrieving the medical record of all patients <18 years of age hospitalized for pH1N1 in the first 3 months of the pandemic from 1 July 2009 through 30 September 2009 and that of age- matched controls hospitalized in our hospital for seasonal influenza H1N1 and H3N2 in the preceding years in a 1∶1 ratio. These age-matched controls were selected patients from the database in the Department of Microbiology according to admission dates closest to the study period, matched to the closest year of age. To overcome the potential bias of comparison with historic controls, we also performed comparisons of children admitted with pH1N1 with children admitted for seasonal influenza during the pandemic period. Parameters collected included underlying conditions deemed high risk of influenza complications, discharge diagnoses, duration of hospitalization, intensive care admission, death, height of fever, white blood count and chest x ray abnormalities and oseltamivir and antibiotic use.

The study protocol was approved by the joint institutional review board of the University of Hong Kong and Queen Mary Hospital (Hong Kong) which waived the written consent. Written consent was not felt to be needed since virologic testing was a routine diagnostic procedure and patient information saved at the study database was delinked from individual patient identification. From the emergence of the pandemic until the 1^st^ of July, Hong Kong implemented a containment and mitigation strategy where all patients confirmed to be infected with pH1N1 were hospitalized and discharged according to isolation and infection control measures, regardless of clinical indication. Starting June 29, 2009, when pH1N1 became more widely circulating in the community, the Department of Health and the Hospital Authority of Hong Kong implemented an adjustment of policy to hospitalize patients on the basis of clinical need rather than recommend mandatory hospitalization of all infected individuals [19]. Therefore, patients for this study were recruited from the 1^st^ July onwards to avoid patients admitted for public health containment purposes irrespective of clinical need. Children were hospitalized either because 1) their respiratory infection symptoms warranted admission through the Emergency Department or 2) their respiratory symptoms led to pH1N1 testing in the outpatient clinics and admitted when they tested positive. All hospitalized children with virologically diagnosed pH1N1 or seasonal influenza were included in the analysis.

NPA were tested for Influenza A and B by direct antigen detection and virus culture in all recruited patients. All the NPA samples were tested for influenza virus type A and B and subtyped as pH1N1, seasonal H3N2 and seasonal H1N1 viruses by RT-PCR as previously described [20]–[21], [22].

Comparisons between children with pandemic influenza A H1N1, seasonal H1N1 and H3N2, as well as that between those with pH1N1 and concurrently admitted seasonal influenza cases during the pandemic period were made. Children with pH1N1 were also compared to all control children admitted for seasonal influenza. Paired t-test was used to detect the difference of continuous variables such as maximum temperature, length of hospital stay between pairs of observation. McNemar's test was used to detect the agreement of the paired proportion in categorical risk factors. Unpaired t-test was used for comparison when there was a missing continuous variable. Chi-square test was used to detect the association of abnormal chest xray, lymphocyte and neutrophil counts between groups when there were missing data in these variables. A p value less than 0.05 was considered statistically significant. Post-hoc analysis demonstrated that 80 subjects per group had 80% power of detecting a 5% difference with a significance level of 0.05.

## Results

Ninety-nine children with a median age of 5.7 (0.4–17.4) years were hospitalized and confirmed to have pH1N1 infection in the 2 study months, with a male to female ratio of 1.25∶1. Since these children were not admitted for mandatory isolation under the containment and early mitigation phases, only 19.2% were known to have pH1N1 on admission, the others were admitted for respiratory symptoms and diagnosed as pH1N1 after admission. During the same time, 37 children with a median age of 2.7 years (6 days to11 years) were hospitalized for seasonal influenza (4 with seasonal H1N1 and 33 with H3N2). The mean age of children with seasonal influenza were younger than those with pH1N1: mean age of 3.6±2.9 years compared to that of 6.6±4.8 years in those with pH1N1 (p = 0.0005).

Patients admitted for pH1N1 infection were more likely to have at least one high risk condition for influenza. (Table 1). There was no difference in clinical diagnosis between the 4 groups of patients with the exception that asthma exacerbation and acute otitis media were diagnosed more often in children admitted for pH1N1 than children with seasonal influenza. (Table 2). Pneumonia was diagnosed in 11.1% of patients infected with pH1N1, 10 % and 11% in age matched children with seasonal H1N1 or H3N2 respectively, and in 5.4% in concurrently admitted children with seasonal influenza (p = NS) and 5.1%, 0%, 3% and 0% of them respectively were thought to have bacterial pneumonia (p = NS). There was no death or intensive care admission in any child in this study.

**Table 1:** Comparisons of pre-existing high risk conditions of children hospitalized for influenza A pH1N1 with age- matched controls hospitalized for seasonal H1N1 and H3N2, and with children hospitalized for seasonal influenza concurrently during the study period, respectively.

	pH1N1	H1N1	H3N2	Concurrent	pH1N1	pH1N1	pH1N1	pH1N1
	(n = 99)	(n = 99)	(n = 99)	seasonal	vs.	vs.	vs.	(N = 99)
				influenza	H1N1	H3N2	concurrent	vs
				(n = 37)	P value	P value	seasonal	all influenza
							influenza	(N = 235)
							P value	
≥1 underlying	40	23	23	7 (18.9%)	0.0125	0.0171	0.032	0.0014
condition	(40.4%)	(23.2%)	(23.2%)					
Prematurity	5	4	7	0	NS	NS	NS	NS
	(5.1%)	(4%)	(7.1%)					
Pulmonary	20	8	10	0	NS	NS	0.007	NS
	(20%)	(8.1%)	(10.1%)					
Cardiac	2	3	1	3 (8.1%)	NS	NS	NS	NS
	(2.0%)	(3.0%)	(1.0%)					
Immunodeficiency	4	4	1	1 (2.7%)	NS	NS	NS	NS
	(4.0%)	(4.0%)	(1.0%)					
Malignancy	1	0	2	1 (2.7%)	-	NS	NS	NS
	(1.0%)		(2.0%)					
Neurologic	10	5	7	2 (5.4%)	NS	NS	NS	NS
	(10.1%)	(5.1%)	(7.1%)					
Renal	2	2	1	0	NS	NS	NS	NS
	(2.0%)	(1.1%)	(1.0%)					
Metabolic	2	1	1	1 (2.7%)	NS	NS	NS	NS
	(2%)	(0.5%)	(0.5%)					

**Table 2:** Comparison of clinical diagnosis of children hospitalized for influenza A pH1N1 with age- matched controls hospitalized for seasonal H1N1 and H3N2, and with children hospitalized for seasonal influenza concurrently during the study period, respectively.

	pH1N1	H1N1	H3N2	Concurrent	pH1N1	pH1N1	pH1N1 vs.	pH1N1
	(N = 99)	(N = 99)	(N = 99)	seasonal	vs.	vs.	concurrent	(N = 99)
				influenza	H1N1	H3N2	seasonal	vs
				(N = 37)	P value	P value	influenza	all
							P value	influenza
								(N = 235)
Uncomplicated	54	50	52	27	NS	NS	NS	NS
URI	(54.5%)	(50.5%)	(52.5%)	(73.0%)				
Febrile seizure	6/44	10/44	13/44	4/23	NS	NS	NS	NS
(6 months-5	(14.0%)	(22.7%)	(29.5%)	(17.4%)				
years)								
Convulsion	11	12	10	1	NS	NS	NS	NS
	(11.1%)	(12.1%)	(10.1%)	(2.7%)				
Encephalopathy/	1	1	0	0	NS	-	NS	NS
encephalitis	(1.0%)	(1.0%)						
Stroke	1	0	0	0	-	-	NS	-
	(1.0%)							
Delirium/	0	1	1	0	-		-	-
confusion		(1.0%)	(1.0%)					
Syncope	1	1	2	0	NS	-	NS	NS
	(1.0%)	(1.0%)	(2.0%)					
Pneumonia (all)	11	10	11	2	NS	NS	NS	NS
	(11.1%)	(10.1)	(11.1%)	(5.4%)				
Bacterial	5	0	3	0	-	NS	NS	NS
pneumonia	(5.1%)		(3.0%)					
Croup	2	3	1	0	NS	NS	NS	NS
	(2.0%)	(3.0%)	(1.0%)					
Asthma	8	1	1	0	0.0455	0.0455	-	0.0014
exacerbation	(8.1%)	(1.0%)	(1.0%)					
Acute	0	1	3	0	-	-	-	NS
bronchiolitis		(1.0%)	(3.0%)					
Acute otitis	5	0	0	2	-	-	NS	0.0425
media	(5.1%)			(5.4%)				
Tonsillitis/	0	1	1	0	-	-	-	-
pharyngitis		(1.0%)	(1.0%)					
Gastroenteritis	1	6	2	1	NS	NS	NS	NS
	(1.0%)	(6.1%)	(2.0%)	(2.7%)				
Viral rash/	0	1	2	0	-	-	-	-
urticaria		(1.0%)	(2.0%)					
Myositis	2	1	0	0	NS	-	NS	NS
	(2.0%)	(1.0%)						

The mean maximum recorded temperature of patients infected with pH1N1 was statistically significantly lower than those infected with seasonal H1N1 and H3N2. Although statistically significant, the clinical significance of mean maximal recorded temperatures of 39.5 vs. 39.7 may be questionable (Table 3) A significant proportion of children infected with any influenza A infection were lymphopenic with no significant difference between pandemic and seasonal influenza. Duration of hospitalization of children with pH1N1 (mean of 3.4±2.1 days) was no different to those concurrently admitted with seasonal influenza but was significantly longer than the 2.5±1.1 days of those in the age matched group with seasonal H1N1 (p = 0.0002). However, only 9.1% of patients with pH1N1 were still febrile at discharge, as compared to 23.2% of those with seasonal pH1N1 (p = 0.0176) and 21.2% with H3N2 (p = 0.031).

**Table 3:** Comparison of duration of fever, investigation results and treatment of children hospitalized for influenza A pH1N1 with age- matched controls hospitalized for seasonal H1N1 and H3N2, and with children hospitalized for seasonal influenza concurrently during the study period, respectively.

	pH1N1	H1N1	H3N2	Concurrent	pH1N1	pH1N1	pH1N1	pH1N1
	(N = 99)	(N = 99)	(N = 99)	Seasonal	vs.	vs.	vs.	(N = 99)
				Influenza	H1N1	H3N2	concurrent	vs
				(N = 37)	P value	P value	seasonal	all
							influenza	influenza
							P value	(N = 235)
Mean days of	2.2±1.5	2.6±2.9	2.2±1.5	2.0±1.54	NS	NS	NS	NS
fever at								
presentation								
Mean days of	3.7±1.8	4.1±3.1	3.9±2.0	3.7±1.5	NS	NS	NS	NS
fever before								
discharge								
% febrile at	9 (9.1%)	23 (23.2%)	21 (21.2%)	4 (10.8%)	0.0176	0.031	NS	0.0185
discharge								
Mean T_max_	39.51±0.59	39.73±0.62	39.85±0.66	39.72±0.65	0.011	0.002	NS	0.0004
(^0^C)								
Mean days of	3.4±2.1	2.5±1.1	2.9±1.6	3.1±2.3	0.0002	NS	NS	NS
hospitalization								
Abnormal	31/83	26/72	39/77	11/33	NS	NS	NS	NS
CXR	(44.6%)	(36.1%)	(50.6%)	(33.3%)				
Lymphocyte <	33/79	36/85	43/96	13/33	NS	NS	NS	NS
1.0×10^9^/L	(41.8%)	(42.4%)	(44.8%)	(39.4%)				
Neutrophil <	3/79	3/85	2/96	3/33	NS	NS	NS	NS
1×10^9^/L	(3.8%)	(3.5%)	(2.1%)	(9.1%)				
Neutrophil >	10/79	13/85	21/96	1/33	NS	NS	NS	NS
8.3×10^9^/L	(12.7%)	(15.3%)	(21.9%)	(3.0%)				
Full course of	50 (50.5%)	0	0	3 (8.1%)	<0.0001	<0.0001	<0.0001	<0.0001
Oseltamivir								
Full course of	18 (18.2%)	14 (14.1%)	13 (13.1%)	5 (13.5%)	NS	NS	NS	NS
antibiotic								

Almost half of the patients admitted for pH1N1 were treated with a full course of oseltamivir, significantly more so that those with seasonal influenza admitted concurrently or in the age matched control group (Table 3). The increased use of oseltamivir in pH1N1 infected children was influenced more by concerns and guidelines of clinical care arising as a result of the pandemic, i.e. patients with underlying health conditions should have antiviral therapy, in contrast to the practice in previous years. We compared pH1N1 infected children who received oseltamivir with similarly infected children who did not. pH1N1 infected children treated with oseltamivir were older with a mean age of 7.8±4.7 years vs. 5.3±4.7 years (p = 0.0095), had a longer duration of hospitalization of 4±2.4 days vs. 2.7±1.4 days (p = 0.0014), and were more likely to have pneumonia: 20.4% vs. 2% (p = 0.0095) when compared to those who did not receive oseltamivir. There was a trend that patients who received oseltamivir were more likely to have one or more high risk conditions. Three of 49 (6.1%) of children who received oseltamivir for pH1N1 had adverse events: one each with vomiting, urticaria and behavioral problems. The child with behavioral changes was a 6 year old boy with moderate asthma attack and discharged from our hospital on oseltamivir. After taking 4 doses of oseltamivir, he became irritable and exhibited some violent acts including pointing a knife at his own neck and hitting his mother. When brought back to the hospital he was alert and conscious but uncooperative for examination. Oseltamivir was withheld, his behavior became normal in the ward and he was discharged the next day.

When age-matched controls with seasonal H1N1 or H3N2 who did not receive oseltamivir were compared to the 49 children with pH1N1 who did not receive oseltamivir, there was no difference in underlying risk conditions or discharge diagnoses. Uncomplicated URI accounted for 63%, 49% and 55% of the diagnoses in children with pH1N1, seasonal H1N1 and H3N2, respectively. (p = NS) Children with pH1N1 had a lower maximum temperature recorded when compared to those with seasonal influenza H3N2.

## Discussion

Disease manifestations seen in children hospitalized with pH1N1 in our patient population was mild. One Canadian study compared 58 children hospitalized with pH1N1 with 200 children infected with seasonal influenza. [17] They found that those with pH1N1 with a median age of 6.6 years were significantly older than those with seasonal influenza A with a median age of 3.3 years. Similar proportions of children required intensive care admission and intubation and the authors concluded that pH1N1 did not appear to cause more severe disease than seasonal influenza A.

There was no death or intensive care admission in any of the hospitalized children in the 4 groups. Only half of the children infected with pH1N1 were treated with oseltamivir. The general recommendation at the time was to offer treatment to all children hospitalized for pH1N1, but the final decision was still up to that of the attending pediatrician in discussion with the parents. Considering that uncomplicated upper respiratory tract infection was the most common single diagnosis, the decision to not treat mild disease is understandable. All except one pH1N1 patients with pneumonia were given oseltamivir and 62.5% of children with one or more high risk conditions were treated as well. The recommendation for any child hospitalized for influenza has now changed to that antiviral should be considered. [23]

In contrast to early reports in adults, gastrointestinal symptoms were not prominent in our group of children hospitalized for pH1N1, regardless of oseltamivir therapy.

Lymphopenia had been associated with pneumonia and respiratory failure in adults. [2], [24] Relative lymphopenia with or without increased monocyte percentage has even been proposed as a surrogate marker of pH1N1 and lymphopenia is thought by some to be uncommon in children with influenza A. [25], [26] However, lymphopenia <1×10^9^/ml was quite commonly seen in our children with uncomplicated pH1N1 infection as well as in children with seasonal influenza.

A significant proportion of children with seasonal H1N1 and H3N2 in years prior to the pandemic were discharged while still febrile in our hospital, likely contributable to the availability of rapid virus diagnosis which reassured parents and doctors of a clinical diagnosis. We have previously shown that availability of rapid virus diagnosis shortens the duration of hospitalization.[27] However, children with pH1N1 and children with seasonal influenza hospitalized during the pandemic period were significantly less likely to be discharged while still febrile, presumably due to a more conservative attitude of both health care workers and parents during a pandemic with an increased reluctance to discharge a child who was still febrile. This low proportion of febrile children at discharge coupled with a lack of difference in duration of fever before and during hospitalization between pH1N1 and seasonal influenza A likely demonstrate that the total duration of fever in children infected with pH1N1 may in fact be shorter than those infected with seasonal influenza.

Up to the end of the study period, that is, as of September 27, 2009, 26,548 people in Hong Kong were virologically confirmed to be infected. [28] Between May 1, 2009 and May 23, 2010, there were a total of 36,546 laboratory-confirmed pH1N1 cases reported to the Centre for Health Protection in Hong Kong. Children aged below 10 years had the highest incidence rate of 2424 cases/100,000 population. Seventeen percent of all laboratory-confirmed cases required hospitalization and children below 5 years of age accounted for 32.8% of these. Public hospitals (of which Queen Mary Hospital is one) cater for 72.5% of general pediatric hospitalizations and almost 100% of PICU hospitalizations in Hong Kong. There has been in place a computerized medical system of all the public hospitals for mandatory reporting of Avian/pandemic influenza admissions (eFlu) since November 2005 and revised in July 2006 [29].Only 13 reports of serious pH1N1 admissions in children <18 years were made in the 5 months of the pandemic which included 11 PICU admissions. There were only 3 pediatric death cases, 2 of these children had cerebral palsy and one child had congenital heart disease in the whole of Hong Kong SAR during that time. The manifestations of pH1N1 in children appeared mild in Hong Kong, and the low case fatality rate corroborated with the findings of this study.

There are strengths and limitations in this study. There is a potential bias of using historical controls for comparison. However this approach was chosen because it was important to age stratify the patients for meaningful clinical comparisons. Using patients with concurrent seasonal influenza does not provide an age matched group for comparison and previous studies using concurrent controls are open to criticism for this reason. Thus we used both age matched historical controls as well as concurrent controls with seasonal influenza for comparison. The observation that pH1N1 is not clinically different to seasonal influenza is supported by both age matched and concurrent control groups. Another potential problem is that the threshold for admission may be different during pandemic and inter-pandemic periods. There might be potential bias for admission to include less severely affected children during the pandemic period who would otherwise not be admitted. This may be true to some extent in Hong Kong as in other places, however, there was no death case or ICU admission in any group in the study. Moreover, the study period was chosen to avoid the period of attempted containment when patients with pH1N1 were admitted for infection control purposes, irrespective of clinical need. Over 80% of the children admitted for pH1N1 were admitted through the Emergency Department and diagnosed after admission. The study was conducted over a short period but it included the period at which pH1N1 peaked in late September and provided us with an adequate number of patients for analysis.

In conclusion, using both age- matched patients with seasonal influenza supplemented with concurrent admissions for seasonal influenza, we demonstrated that children hospitalized for pH1N1 did not have more severe disease when compared to their controls infected with seasonal H1N1 or H3N2, although they were more likely to have one or more conditions deemed at risk for severe influenza infection, a chronic pulmonary condition, more likely to have asthma exacerbation and they were more likely to receive oseltamivir, especially if they had pneumonia. Overall, disease manifestations of children hospitalized for pH1N1 infection in our patient population were mild.
